# Supplementary material for: Analysis of PPARγ Signaling Activity in Psoriasis
Source: Int J Mol Sci. 2021 Aug 10;22(16):8603. doi: 10.3390/ijms22168603 (PMC8395241; doi:10.3390/ijms22168603)
Supplement: Supplementary file 1 [file ijms-22-08603-s001.zip › Supplemental materials_Analysis of PPARg signaling activity in psoriasis/Pathway models/Models images and html files/PPARG inhibits phenotypes assotiated with psoriasis/210771.html]

sensitization


# Cell Process sensitization

|  |  |
| --- | --- |
| URN | urn:agi-gocellproc:0046960 |
| Total Entities | 3 |
| Connectivity | 4998 |
| Name | sensitization |

---

|  |  |
| --- | --- |
| ChildConcepts | chemosensitization |
|  | contact sensitization |
|  | radiosensitization |

---

|  |  |
| --- | --- |
| Pathway | PPARG inhibits phenotypes assotiated with psoriasis |
|  | efects selected |
|  | Neighbors of sensitization |

---

|  |  |
| --- | --- |
| MedScan ID | 4218816 |

---

|  |  |
| --- | --- |
| Alias | sensibilization |
|  | sensitization |
|  | sensibilizations |
|  | sensitizations |

---

|  |  |
| --- | --- |
| GO ID | 0046960 |

---
